# The Impact of Noise Pollution on Cognitive Function in Middle-Aged and Older Adults: Empirical Evidence from the CHARLS

**DOI:** 10.3390/bs15101404

**Published:** 2025-10-16

**Authors:** Yanzhe Zhang, Yushun Han, Kaiyu Guan

**Affiliations:** 1 Northeast Asian Studies College, Jilin University, Changchun 130012, China; yanzhe_zhang@jlu.edu.cn (Y.Z.); hanys22@mails.jlu.edu.cn (Y.H.); 2 Northeast Asian Research Center, Jilin University, Changchun 130012, China; 3 Peking University Institute of Mental Health, Beijing 100083, China

**Keywords:** noise pollution, cognitive function, sleep, depressive symptoms, CHARLS

## Abstract

Against the backdrop of rapid population aging and a high prevalence of cognitive impairment in China, identifying modifiable environmental risk factors is a public health priority. Although environmental noise is widely recognized as a significant stressor, its effects on cognitive health remain underexplored within the Chinese context. Drawing on balanced panel data from three waves of the China Health and Retirement Longitudinal Study (CHARLS), we examined 3459 individuals aged 45 and above to assess the association between noise pollution and cognitive function using a two-way fixed-effects model. Additionally, we employed a chained mediation approach to investigate whether sleep disturbances and depressive symptoms serve as intermediary mechanisms. The findings indicated a significant inverse relationship: each unit increase in the noise pollution index corresponded to a 0.41-point reduction in overall cognitive scores. These results were robust across various noise exposure measures. Sensitivity analyses using alternative noise metrics also supported this finding. Sleep duration and depression were identified as significant mediators in the relationship between noise pollution and cognitive decline. This longitudinal analysis offers compelling evidence that environmental noise constitutes a substantial risk factor for declining cognitive function in middle-aged and older adults in China.

## 1. Introduction

In light of China’s rapidly aging population, increasing focus has been directed toward safeguarding cognitive health in older adults. By the end of 2024, the population of people 60 and older increased to 310.3 million, representing 22.0% of the national population (National Bureau of Statistics of China, 2025). Concurrently, accumulating evidence suggests that China harbors the largest global population of older individuals experiencing cognitive impairment. The prevalence of mild cognitive impairment (MCI) in this demographic has reached 15.5%, a rate notably exceeding global averages reported across various countries (Huang et al., 2025; Jia et al., 2020). MCI is generally regarded as an intermediate stage between normative cognitive aging and dementia, with approximately 10–15% of affected individuals advancing to dementia each year—most commonly Alzheimer’s disease (Vega & Newhouse, 2014; Wang et al., 2023). Preserving cognitive function is essential for enabling independent living and promoting active social engagement. Its deterioration not only diminishes quality of life and impairs daily functioning in older adults (Tsang et al., 2025), but also imposes significant demands on families and healthcare infrastructures. More importantly, existing interventions for cognitive impairment remain limited and often prove unsatisfactory. In light of China’s irreversible demographic aging, identifying the risk factors that affect cognitive function in later life is essential for enabling early intervention and informing targeted policy responses.

Environmental noise is one of the major environmental hazards to human health, according to the World Health Organization (2024). Growing research supports the view that prolonged or high-intensity exposure to noise can detrimentally impact both physical and psychological well-being (Hahad et al., 2025). Research findings suggest that these detrimental effects extend beyond auditory damage, encompassing a range of non-auditory health outcomes such as increased risks for cardiovascular conditions (e.g., hypertension, ischemic heart disease, Braithwaite et al., 2019; Hahad et al., 2023; Münzel et al., 2018), disruptions to sleep, and elevated levels of anxiety and depression (Jin et al., 2024; Leger et al., 2024). In recent years, increasing scholarly attention has been directed toward concerns about China’s noise environment (Ren et al., 2024; Zhou et al., 2023). Recent statistics indicate that approximately 45% of environmental complaints in China in 2021 were related to noise pollution, making it the second most pressing environmental issue after air pollution (Ministry of Ecology and Environment of the People’s Republic of China, 2022). According to the 2022 Bulletin on the State of the Ecological Environment, the average daytime noise level in Chinese urban areas was 54.1 dB, with fewer than 5% of cities—just 16 nationwide—meeting Level I acoustic quality benchmarks (Ministry of Ecology and Environment of the People’s Republic of China, 2023). Against this backdrop, a critical question arises: What effect does noise pollution have on middle-aged and older persons’ cognitive function in China?

A growing corpus of research has investigated the cognitive hazards linked to increasing levels of environmental noise. Accumulating evidence suggests that prolonged exposure to ambient noise may accelerate cognitive aging. Weuve et al. (2021) observed that each 10-decibel increase in community noise increased the probability of mild cognitive impairment by 36% and Alzheimer’s disease by 29% among Chicago’s 65-year-olds. These findings suggest noise pollution may cause cognitive deterioration. However, the association remains contested, as several studies have not observed statistically significant relationships between exposure to noise and cognitive function. For instance, Andersson et al. (2018), drawing on Swedish data, reported no significant connection between noise levels exceeding 55 dB (Leq. 24 h) and dementia risk. Likewise, Mac Domhnaill et al. (2021) found no correlation between noise exposure and memory or processing speed in Irish adults. These divergent findings may stem from variations in study design (e.g., cross-sectional), exposure measurement techniques (e.g., self-reported versus modeled noise levels), cognitive assessment tools, sample characteristics (e.g., age, baseline health, socioeconomic status), and the extent to which potential confounding variables were controlled (Thompson et al., 2022; Tzivian et al., 2015).

Although prior research has explored the link between noise exposure and cognitive function, the evidence is still scant. First, the majority of studies to date have tended to concentrate on North America and Europe, with an insufficient focus on developing nations, particularly China, which is undergoing rapid urbanization and profound demographic shifts. Given the cross-national differences in noise characteristics, sociocultural contexts, and health systems, it is difficult to generalize findings from Western countries to the Chinese context. Second, although it is widely believed that noise affects cognition through multiple pathways, few studies have investigated these mediating mechanisms in any systematic way. Noise may impair cognitive function through both direct pathways, such as distraction and cognitive load, and indirect ones. For example, noise may act as a chronic stressor that elicits both physiological and psychological responses (Zekveld et al., 2011). Recent research by Leger et al. has shown that noise exposure impairs sleep quality, which is widely recognized as an important cause of cognitive decline (Hu et al., 2021). Researchers, including Jin et al. (2024), have shown that extended exposure to noisy surroundings increases the incidence of depression, a major predictor of cognitive deterioration. However, very little is currently known about whether noise affects cognition through a sequential pathway involving sleep disturbance and depression. Clarifying these complex mechanisms is essential for developing a more complete understanding of how environmental noise may contribute to cognitive health decline.

This study aims to further existing research by methodically investigating the cognitive effects of exposure to environmental noise in the Chinese context. We examine noise pollution and cognitive performance in middle-aged and elderly Chinese people, utilizing data from the CHARLS waves of 2015, 2018, and 2020. Additionally, the study investigates whether sleep duration and depressive symptoms act as sequential mediators, thereby shedding light on the psychological and physiological mechanisms through which noise may impair cognition. Another aim is to identify subgroups that may be particularly susceptible to the adverse cognitive effects of environmental noise. Grounded in established theoretical models and prior empirical research, two hypotheses are proposed. First, based on prior evidence indicating a negative association between environmental noise and cognitive function, Hypothesis 1 (H1) posits that individuals chronically exposed to elevated noise levels will exhibit accelerated cognitive decline or poorer cognitive performance, after accounting for potential confounders. Hypothesis 2 (H2) proposes a chained mediation effect in which sleep and depression jointly mediate the noise–cognition link. Specifically, we hypothesize three indirect pathways: (a) noise → reduced sleep → cognitive decline; (b) noise → increased depression → cognitive decline; and (c) noise → reduced sleep → increased depression → cognitive decline.

## 2. Materials and Methods

### 2.1. Participants

This study centers on middle-aged and older adults included in the CHARLS. Launched in 2011, the CHARLS is an ongoing panel survey that spans 150 counties and 450 communities nationwide. Its primary purpose is to evaluate the health, economic circumstances, and social conditions of individuals aged 45 years and older. The database contains detailed information on physical health, mental health, and sociodemographic characteristics, including indicators like depressive symptoms, cognitive function, and mental status. The CHARLS is known for its extensive coverage, strong representativeness, and reliable data, making it widely used in research studies (Cui et al., 2024; Huo et al., 2023).

**Figure 1:**
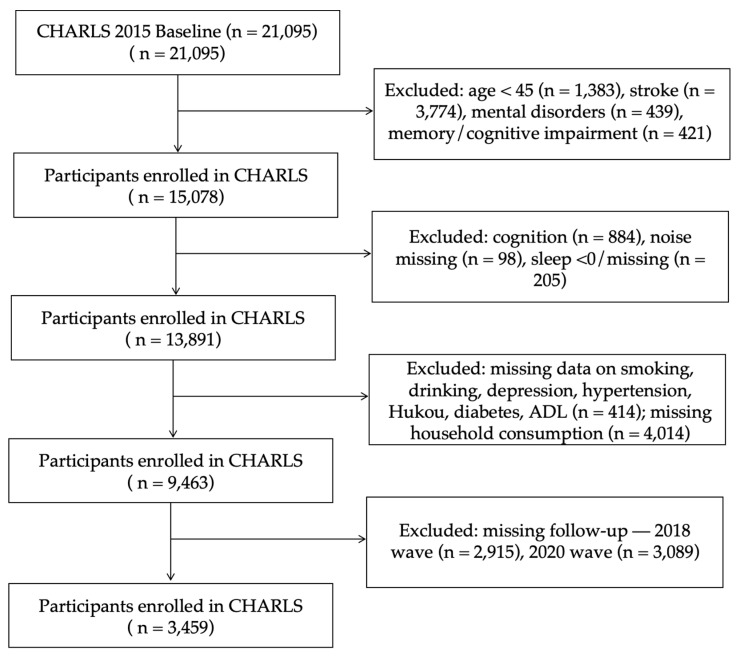
Flow chart of participant selection and exclusion criteria.

This study utilized follow-up data from the 2015, 2018, and 2020 waves of the CHARLS. The 2015 wave was selected as the baseline due to its substantially higher number of follow-up respondents. To isolate the effects of normal aging and minimize confounding by major neuropsychiatric conditions, a series of exclusion criteria were applied to ensure data quality and sample relevance (Tao et al., 2025): (1) 1383 participants have been excluded for lacking age data or being under 45; (2) 3774 participants with stroke, 439 with mental illness, and 421 with memory-related disorders or missing values were excluded; (3) 884 individuals with missing values for cognitive ability and 98 with missing noise exposure data were excluded; an additional 205 participants with invalid (i.e., <0) or missing sleep duration data were also excluded; (4) 414 cases with missing values for smoking, drinking, depression, hypertension, household registration, diabetes, and ADL were excluded; (5) 4014 participants with missing values for total household consumption were excluded; (6) samples with missing follow-up in 2018 (n = 2915) and 2020 (n = 3089) were excluded (Figure 1).

### 2.2. Measures

#### 2.2.1. Cognitive Function

Cognitive performance in the CHARLS dataset was evaluated through three core domains: episodic memory, orientation and attention, and visuospatial skills. Episodic memory was examined via a word recall exercise. Participants were instructed to immediately recall as many of 10 orally presented Chinese words as possible, in any sequence (immediate recall), followed by a second recall attempt after a 4 min delay (delayed recall). Following established research conventions, we derived the episodic memory score by averaging the immediate and delayed recall results, yielding a range from 0 to 10. Orientation and attention were measured using the Telephone Interview for Cognitive Status (TICS), as implemented in CHARLS. This instrument includes 10 items that require participants to report the current date (year, month, and day), day of the week, season, and to perform serial subtractions of 7 from 100. The resulting TICS score, ranging from 0 to 10, reflected the number of accurate responses. Visuospatial functioning was assessed through a figure replication task. Participants were shown an image consisting of two intersecting pentagons and asked to reproduce it. A correct replication was scored as 1, and an incorrect one as 0 (Guo & Zheng, 2023; Kesavayuth et al., 2018; Ma et al., 2016).

#### 2.2.2. Noise Pollution

The assessment of noise pollution was based on search volumes of noise-related keywords obtained from the Baidu Index, a data-sharing platform built on the extensive behavioral data of Baidu’s internet users. The Baidu Index tracks and records the frequency of keyword searches and converts these into a standardized “search index,” with data derived separately from PC and mobile platforms (Higgins et al., 2020; Xiao et al., 2023). In our analysis, the two categories were combined to create a total search index. The Baidu Index has been widely used in recent studies as a proxy for public attention to social and environmental issues. Its advantage lies in capturing dynamic variations in public concern both temporally and spatially. In general, higher search indices for noise-related terms indicate greater public concern and complaints about noise, which indirectly reflect higher levels of local noise intensity. Prior research has confirmed that this measure provides a meaningful proxy for assessing environmental noise trends. Following Xiangli et al. (2025), two Chinese keywords—zao sheng and zao yin—were selected to represent noise pollution. Daily search indices of these keywords were collected for 125 cities across China in 2013, 2015, and 2018. For each city, the annual sums of the daily search indices were calculated and then log-transformed to generate a city-level “noise index.” This index served as the primary independent variable in the subsequent analyses.

#### 2.2.3. Covariates

Informed by previous studies on the determinants of cognitive function, the present study included additional covariates (Wu et al., 2024; Zhang & Jiang, 2022). These included demographic characteristics (age, sex, marital status), socioeconomic indicators (educational level, Hukou status, which refers to China’s household registration system that classifies individuals as either urban or rural residents), physical health measures (activities of daily living [ADL], hypertension, diabetes), behavioral factors (smoking and alcohol consumption), and economic status as reflected by per capita household consumption.

#### 2.2.4. Mediated Variables

The 10-item shortened Center for Epidemiologic Studies Depression Scale (CES-D) was used to assess depressed symptoms concisely and reliably. Participants scored on a four-point scale: “rarely or none of the time = 0, “some or somewhat = 1, “sometimes or a considerable amount = 2,” and “most or all of the time = 3.” Summing item scores yielded the CES-D score, which ranged from 0 to 30 (Carlson et al., 2011).

Sleep duration was measured using a self-reported item asking, “How many hours of actual sleep did you receive during the past month?” This measure was incorporated as a continuous variable in the analytical models (Fan et al., 2025).

### 2.3. Statistical Analysis

We used a two-way fixed effects panel regression model to examine the connection between noise pollution and cognitive function. This approach controls for both individual-level and time-specific unobserved heterogeneity, thereby reducing potential bias in the estimated effects. This modeling approach is particularly useful in controlling for unobserved heterogeneity and macro-level temporal shocks, thereby reducing omitted variable bias and producing more reliable estimates. The specific form of the model can be expressed as follows:

(1) $${Cognitive}_{it}=\beta_{0}+\beta_{1}{Noise}_{it}+\beta_{2}X_{it}+\delta_{i}+\mu_{t}+\varepsilon_{it}$$

In this equation, ${Cognitive}_{it}$ denotes the cognitive function score of respondent *i* in period *t*; ${Noise}_{it}$ refers to the noise pollution index of the city where respondent *i* resides in period *t*; and $X_{it}$ represents a set of covariates. $\delta_{i}$ denotes individual fixed effects that control for time-invariant respondent-specific characteristics; $\mu_{t}$ refers to time fixed effects that capture macro-level shocks common to all respondents in period *t*; and $\varepsilon_{it}$ is the idiosyncratic error term.

Additionally, sensitivity analyses were carried out using alternative noise measures, including the Environmental Quality Standard for Noise and road traffic noise levels. Categorical variables were summarized using frequencies and percentages [n (%)], with group comparisons assessed via chi-square (χ^2^) tests. Data were reported as means with standard deviations ($\bar{x}$ ± s) for continuous variables, and intergroup comparisons used independent samples t-tests. Statistical significance was set at *p* < 0.05. All statistical methods were run in R 4.2.1.

## 3. Results

### 3.1. Baseline Characteristics of Participants

The final analytical sample comprised 3459 middle-aged and older adults. Among them, 1692 participants (55.65%) were men, while 1534 (44.35%) were women. The average age of the sample was 59.14 years (SD = 7.94). The average depression score was 7.10 (SD = 5.80), while the mean reported sleep duration was 6.42 h per night (SD = 1.67). The majority of participants (92.25%) were married, and 61.55% held rural household registration status. Additionally, 30.44% reported current smoking, 39.72% had a diagnosis of hypertension, and 9.31% reported having diabetes.

**Figure 2:**
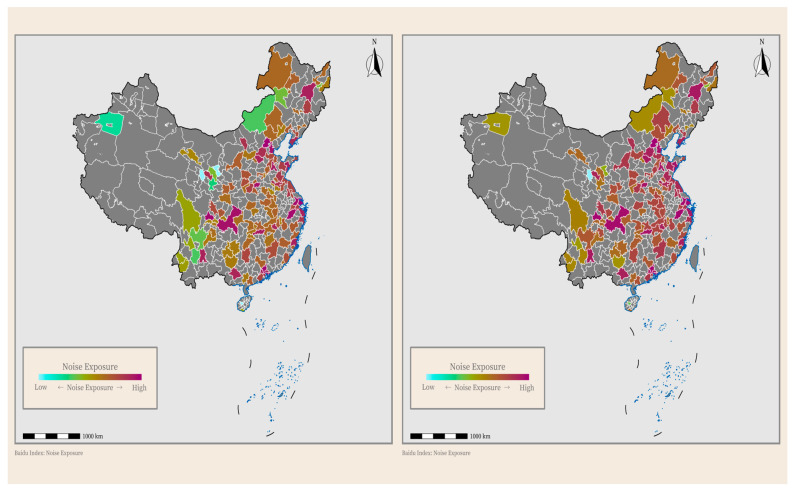
Spatial distribution of noise in 2015 (**left**) and 2020 (**right**).

Participants were categorized into two groups based on the median value of environmental noise exposure: the low-exposure group (noise pollution ≤ 8.268) and the high-exposure group (noise pollution > 8.268). Descriptive analyses indicated statistically significant differences (*p* < 0.05) between these groups in several variables, including age, depressive symptoms, education level, Hukou, smoking, and diabetes prevalence. Summary statistics for these variables are presented in Table 1. Additionally, Figure 2 illustrates a clear upward trend in environmental noise levels between 2015 and 2020.

### 3.2. Correlation Analysis

The correlation analysis revealed a statistically significant but weak inverse relationship between noise exposure and cognitive function (r = −0.094, *p* < 0.01). A moderate negative correlation was observed between noise levels and sleep duration (r = −0.290, *p* < 0.01). Additionally, noise pollution showed a weak yet significant positive association with depressive symptoms (r = 0.023, *p* < 0.05) (Table 2).

### 3.3. Results of Multistep Regression Analysis of Noise Pollution on Cognitive Function

Table 3 reports the results from the multivariate linear regression models with individual and time-fixed effects. In Model 1, only the noise pollution variable was included, revealing a significant negative association with cognitive function (β = −0.408, *p* < 0.01). Model 2 introduced gender and age as additional covariates. Model 3 expanded the controls to include demographic characteristics such as marital status, residential type, educational attainment, and household consumption. In Model 4, health-related factors—including diabetes, hypertension, and limitations in activities of daily living—were further adjusted for. Across all four models, noise pollution consistently exhibited a significant negative relationship with cognitive function.

### 3.4. Sensitivity Analysis

First, this study utilized average equivalent sound levels and road traffic noise data from the National Urban Acoustic Environment Quality Report, issued by the China National Environmental Monitoring Center (Ministry of Ecology and Environment of the People’s Republic of China, 2025), to operationalize noise pollution. The regression analyses were re-estimated based on these indicators. As the report covers only provincial capitals and municipalities in China—including 22 cities represented in the CHARLS dataset, accounting for approximately 16.63% of the total sample—and as these cities tend to perform below the national average in terms of compliance rates at functional monitoring points and acoustic quality in both regional and road traffic contexts, the noise measurements derived from this source are considered to be more representative and reliable.

Secondly, a Cox regression analysis was performed using a cognitive impairment outcome variable, defined according to the criteria established by Jak et al. (2016), whereby impairment was identified as a cognitive function score more than 1.5 standard deviations below the sample mean.

Thirdly, this study also employed the Mini-Mental State Examination (MMSE) to measure cognitive function. In the CHARLS 2018 survey, the MMSE scale was used to assess participants’ cognitive status.

Table 4 presents the regression results based on alternative noise exposure indicators. Column 1 reports estimates derived from average equivalent sound levels, while Column 2 displays results based on road traffic noise data. In both cases, the associations remain consistent with the primary findings. Column 3 displays the Cox regression results, revealing that each one-unit increase in environmental noise was linked to a 6.23% elevated risk of cognitive impairment (hazard ratio = 1.0623, 95% CI: 1.016–1.111). These findings align with and further support the conclusions drawn from the main analysis. Column 4 presents the regression results based on MMSE-assessed cognitive function scores, showing that noise pollution remained significantly and negatively associated with cognitive function (β = −0.219, *p* < 0.01).

### 3.5. Analysis of the Mediating Effects of Sleep and Depression

After controlling for all covariates, a panel mediation model was constructed to examine the chained mediating effects of sleep duration and depression on the relationship between noise pollution and cognitive function.

**Figure 3:**
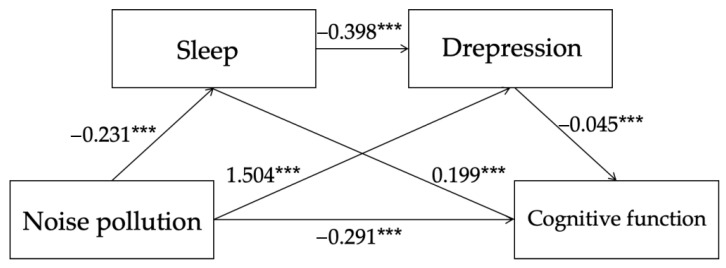
The chain mediation model of depression and sleep between noise pollution and cognitive function. Note: *** indicate significance at the 1% levels.

The analysis indicated that noise pollution had a significant total effect on cognitive function (β = −0.413, *p* < 0.001). Even after incorporating the mediating variables, the adverse impact of noise exposure on cognitive outcomes remained statistically significant (β = −0.291, *p* < 0.001). Additional findings indicated a negative relationship between noise and sleep duration (β = −0.231, *p* < 0.001), and a positive relationship between noise levels and depressive symptoms (β = 1.504, *p* < 0.05). Moreover, sleep duration was inversely linked to depression (β = −0.398, *p* < 0.001) and positively linked to cognitive performance (β = 0.199, *p* < 0.001). The estimated total indirect effect of noise on cognitive performance was −0.122 (95% CI: −0.150 to –0.096), accounting for 29.52% of the overall effect. The remaining 70.48% was attributable to the direct pathway (β = −0.291, 95% CI: −0.443 to −0.146). A full summary of the results is provided in Figure 3 and Table 5.

### 3.6. Subgroup Analyses

**Figure 4:**
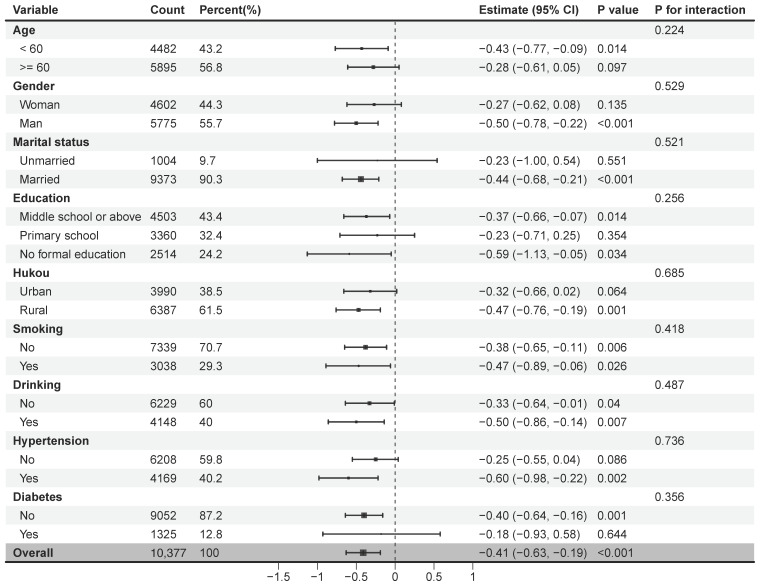
Subgroup analysis.

Subgroup analyses further examined the association between noise exposure and cognitive outcomes by stratifying participants according to demographic and health-related characteristics, including age, gender, marital status, education level, Hukou type, smoking and drinking, and presence of hypertension or diabetes. After adjusting for household consumption and other covariates, the negative relationship between noise exposure and cognitive performance remained statistically significant across most subgroups. Notable exceptions included individuals aged over 60, those who were unmarried, had only completed primary education, or were diagnosed with diabetes, all of whom exhibited comparatively weaker negative effects. These results underscore the broad and robust nature of the adverse cognitive effects of environmental noise, as detailed in the subgroup findings presented in Figure 4.

## 4. Discussion

This study aimed to examine the impact of environmental noise pollution on cognitive function in middle-aged and older adults, utilizing nationally representative longitudinal data from the CHARLS. A secondary objective explored whether sleep duration and depressive symptoms functioned as sequential mediators in this relationship. The findings revealed a significant negative relationship between noise exposure and cognitive performance, indicating that higher noise levels are linked to poorer cognitive performance. These results were further supported by sensitivity analyses, which yielded consistent outcomes when alternative indicators of noise and cognitive measures were applied. These results provide some tentative initial evidence that sleep duration and depression may operate as sequential mediators in the pathway from noise exposure to cognitive impairment. Subgroup analyses suggested that the adverse effects of noise pollution were pervasive across different population groups.

One of the central contributions of this study is the identification of a robust negative association between environmental noise and cognitive performance in China’s aging population. This result aligns with prior research conducted in high-income nations, where extensive epidemiological evidence has linked prolonged exposure to environmental noise—particularly traffic-related noise—to elevated risks of cognitive deterioration, mild cognitive impairment, and even dementia (Jak et al., 2016; Mac Domhnaill et al., 2021). The effect size was comparable to hypertension and to several years of natural aging, underscoring the public health importance of noise as a modifiable risk factor. Prior investigations have indicated that long-term exposure to ambient noise and air pollutants can accelerate age-related cognitive deterioration (Ogurtsova et al., 2023), and some evidence suggests that these environmental stressors may interact synergistically to amplify their adverse effects on brain health (Yu et al., 2023). Despite these insights, most existing studies have focused on high-income settings, while research from China—a major developing country with a rapidly aging population—remains limited. The current study addresses this gap by leveraging a nationally representative longitudinal dataset from China, offering the first comprehensive examination of how environmental noise may affect cognitive function in this population. Given the complexity of cognitive decline trends in China, incorporating noise—a ubiquitous yet often overlooked environmental risk factor—into the cognitive health research framework holds important practical implications for the development of comprehensive aging strategies and public health policies (Goines & Hagler, 2007; Guo & Zheng, 2023; Hammer et al., 2014).

Noise, as a persistent environmental stressor, may exert its influence on both psychological well-being and physiological processes through several distinct mechanisms. The current findings align with prior research indicating that prolonged exposure to noisy settings can disrupt sleep patterns (Hahad et al., 2023; Halperin, 2014; Kim et al., 2024; Miedema & Vos, 2007). Individuals frequently exposed to noise may experience heightened irritability and anxiety, which can escalate into depressive symptoms over time (Evans, 2021). In line with these observations, existing literature has also identified sleep disruption and depression as significant contributors to cognitive deterioration (Chu et al., 2021; Geng et al., 2025; Hahad et al., 2025; Mehta et al., 2022). These findings provide further support for the hypothesis that noise pollution may impair cognitive function through multiple pathways, including (1) reduced sleep duration and subsequent depression, (2) sleep-mediated effects, and (3) depression-mediated effects. The results shed light on the complex dynamics linking environmental noise and cognitive outcomes, underscoring the sequential mediating roles of sleep and emotional health. This suggests that, beyond its direct influence on cognitive functioning, noise may initiate a cascade of physiological and psychological effects that merit further investigation.

One of the most notable findings is that the negative impact of noise exposure on cognitive performance was especially evident among individuals who initially exhibited higher levels of cognitive abilities. Subgroup analysis showed that, with the exception of certain groups—such as those over 60 years of age, unmarried individuals, those with only a secondary education, or those diagnosed with diabetes, who exhibited comparatively smaller effects—the adverse influence of noise exposure on cognitive outcomes was pervasive across the broader population. These groups often exhibit lower baseline cognitive function and limited cognitive reserve, or greater vulnerability to strong biomedical risk factors (e.g., diabetes, cardiovascular disease). In such cases, the relative contribution of community noise may be overshadowed by stronger competing risks, which could explain the attenuated or non-significant associations we observed. This pattern underscores the widespread nature of noise pollution as a significant public health concern, suggesting that its detrimental effects are not limited to traditionally vulnerable groups. On the contrary, individuals with relatively better baseline cognitive functioning appeared to experience more pronounced cognitive decline when exposed to noisy environments. This further highlights the urgency of incorporating noise pollution control into national public health agendas. In light of accelerating urban development and an aging population, ensuring quieter and healthier living conditions for all residents should be a key component in promoting the overarching objective of healthy aging (Bocancea et al., 2023). Although the observed correlations were modest in magnitude, they are consistent with prior large-scale studies and may still carry meaningful public health implications at the population level.

A note of caution is due here since this study has several limitations. First, the measurement of noise pollution relied on the Baidu Index, which is an indirect indicator. These data must be interpreted with caution because the Baidu Index may be influenced by external factors such as media coverage and environmental awareness, which may result in measurement bias. Although these results were further supported by sensitivity analyses using official noise-level data, the coverage of such data was limited to a subset of cities. Further work is needed to develop more accurate and individualized exposure assessments, such as personal noise monitors or high-resolution noise maps. Second, cognitive function was assessed using simplified tests from the CHARLS survey. Although this method has been widely used in epidemiological research, further work is needed to gain a better understanding of cognitive mechanisms by incorporating more refined tools and detailed evaluation indicators. Third, the analysis of cumulative noise exposure duration and patterns remains pre-liminary. Further work is needed to explore the mechanisms behind long-term cumulative effects and to identify potential critical windows of exposure using long-term follow-up and more detailed exposure assessments. Finally, we may have overlooked certain confounding factors, such as air pollution and lifestyle variables, which should be incorporated in future research to provide a more comprehensive understanding of how multiple environmental and behavioral exposures jointly influence cognitive health.

## 5. Conclusions

This study, drawing on a large and nationally representative longitudinal dataset, identified a significant negative relationship between environmental noise pollution and cognitive function in the Chinese population. The analysis yielded compelling empirical evidence indicating that exposure to noise is significantly associated with reduced cognitive health. This relationship remained consistent even after adjusting for a comprehensive set of confounding variables and employing alternative indicators of both noise exposure and cognitive decline. Notably, the analysis revealed that sleep duration and depressive symptoms jointly served as partial sequential mediators in the pathway from noise pollution to cognitive decline. These insights underscore the critical role of noise management in preserving cognitive health during aging and emphasize the relevance of environmental interventions in public health strategies aimed at supporting healthy cognitive aging.

Looking ahead, research should focus on refining methods for assessing noise exposure, deepening our understanding of the biological and psychological mechanisms involved, and establishing a stronger empirical foundation for developing targeted interventions. Given the accelerating pace of population aging and continuing urbanization, incorporating the cognitive health effects of noise pollution into public health policymaking is of profound significance for building a healthier China.

## Figures and Tables

**Table 1:** Baseline characteristics of participants.

Variable	Total (n = 3459)	Low Exposure (n = 1692)	High Exposure (n = 1767)	Statistic	*p*-Value
Cognitive function score	12.24 ± 3.55	12.39 ± 3.42	12.09 ± 3.67	2.45	0.01
Age (years)	59.14 ± 7.94	58.71 ± 7.90	59.55 ± 7.95	−3.09	<0.01
Depression	7.10 ± 5.80	7.43 ± 5.87	6.78 ± 5.72	3.28	<0.01
Sleep (hours)	6.42 ± 1.67	6.42 ± 1.70	6.41 ± 1.65	0.14	0.89
ADL	0.22 ± 0.69	0.24 ± 0.70	0.21 ± 0.67	1.13	0.26
Consumption	3.32 ± 4.76	3.41 ± 5.38	3.24 ± 4.07	1.02	0.31
Gender				1.18	0.28
Woman	1534 (44.35)	734 (43.38)	800 (45.27)		
Man	1925 (55.65)	958 (56.62)	967 (54.73)		
Marital status				0.00	1.00
Non-married	268 (7.75)	131 (7.74)	137 (7.75)		
Married	3191 (92.25)	1561 (92.26)	1630 (92.25)		
Education				15.53	<0.01
Middle school or above	1461 (42.24)	707 (41.78)	754 (42.67)		
Primary school	826 (23.88)	395 (23.35)	431 (24.39)		
No formal education	1086 (31.40)	530 (31.32)	556 (31.47)		
Hukou				7.47	<0.01
Urban	1330 (38.45)	611 (36.11)	719 (40.69)		
Rural	2129 (61.55)	1081 (63.89)	1048 (59.31)		
Smoking				4.11	0.04
No	2406 (69.56)	1149 (67.91)	1257 (71.14)		
Yes	1053 (30.44)	543 (32.09)	510 (28.86)		
Drinking				1.08	0.30
No	2021 (58.43)	973 (57.51)	1048 (59.31)		
Yes	1438 (41.57)	719 (42.49)	719 (40.69)		
Hypertension				0.06	0.81
No	2085 (60.28)	1016 (60.05)	1069 (60.50)		
Yes	1374 (39.72)	676 (39.95)	698 (39.50)		
Diabetes				6.05	0.01
No	3137 (90.69)	1556 (91.96)	1581 (89.47)		
Yes	322 (9.31)	136 (8.04)	186 (10.53)		

**Table 2:** Correlation analysis.

Variables	(1)	(2)	(3)	(4)
Cognitive function score	1.000			
Depression score	−0.240 ***	1.000		
Sleep duration	0.091 ***	−0.290 ***	1.000	
Noise pollution	−0.094 ***	0.023 **	−0.028 ***	1.000

Note: **, *** indicate significance at the 5%, and 1% levels, respectively.

**Table 3:** Association between noise pollution and cognitive function: multivariate linear regression.

	β	(95%CI)	*p*
Model 1	−0.408	[−0.629, −0.187]	<0.001
Model 2	−0.408	[−0.629, −0.187]	<0.001
Model 3	−0.410	[−0.631, −0.189]	<0.001
Model 4	−0.410	[−0.631, −0.189]	<0.001

**Table 4:** Sensitivity analysis.

	(1)	(2)	(3)	(4)
	Regression	Regression	Cox	Regression
Equivalent sound	−0.379 ***			
Road traffic noise		−0.379 ***		
Noise pollution			1.0623 **	
Noise pollution				−0.219 ***

Note: **, *** indicate significance at the 5%, and 1% levels, respectively.

**Table 5:** Multiple chain mediators.

Effect Type	Effect Value	Boot SE	Boot LLCI	Boot ULCI	Proportion
Total Effect	−0.413	0.076	−0.562	−0.270	
Direct Effect	−0.291	0.076	−0.443	−0.146	70.480
Total Indirect Effect	−0.122	0.014	−0.150	−0.096	29.520
Noise→Sleep→Cognition	−0.046	0.008	−0.063	−0.032	11.149
Noise→Depression→Cognition	−0.072	0.010	−0.091	−0.052	17.371
Noise→Sleep→Depression→Cognition	−0.004	0.001	−0.006	−0.003	1.002

Note: The analytic sample consisted of 3459 participants with complete data across three CHARLS survey waves.

## Data Availability

The datasets presented in this study can be found in online repositories (https://charls.charlsdata.com/).
